# Facial fractures and associated injuries in high- versus low-energy trauma: all are not created equal

**DOI:** 10.1186/s40902-020-00264-5

**Published:** 2020-06-24

**Authors:** Cameron St. Hilaire, Arianne Johnson, Caitlin Loseth, Hamid Alipour, Nick Faunce, Stephen Kaminski, Rohit Sharma

**Affiliations:** Santa Barbara Cottage Hospital, Santa Barbara, California USA

**Keywords:** Facial fractures, High-energy mechanisms, Low-energy mechanisms, Outcomes

## Abstract

**Introduction:**

Facial fractures (FFs) occur after high- and low-energy trauma; differences in associated injuries and outcomes have not been well articulated.

**Objective:**

To compare the epidemiology, management, and outcomes of patients suffering FFs from high-energy and low-energy mechanisms.

**Methods:**

We conducted a 6-year retrospective local trauma registry analysis of adults aged 18–55 years old that suffered a FF treated at the Santa Barbara Cottage Hospital. Fracture patterns, concomitant injuries, procedures, and outcomes were compared between patients that suffered a high-energy mechanism (HEM: motor vehicle crash, bicycle crash, auto versus pedestrian, falls from height > 20 feet) and those that suffered a low-energy mechanism (LEM: assault, ground-level falls) of injury.

**Results:**

FFs occurred in 123 patients, 25 from an HEM and 98 from an LEM. Rates of Le Fort (HEM 12% vs. LEM 3%, *P* = 0.10), mandible (HEM 20% vs. LEM 38%, *P* = 0.11), midface (HEM 84% vs. LEM 67%, *P* = 0.14), and upper face (HEM 24% vs. LEM 13%, *P* = 0.217) fractures did not significantly differ between the HEM and LEM groups, nor did facial operative rates (HEM 28% vs. LEM 40%, *P* = 0.36). FFs after an HEM event were associated with increased Injury Severity Scores (HEM 16.8 vs. LEM 7.5, *P* <0.001), ICU admittance (HEM 60% vs. LEM 13.3%, *P* <0.001), intracranial hemorrhage (ICH) (HEM 52% vs. LEM 15%, *P* <0.001), cervical spine fractures (HEM 12% vs. LEM 0%, *P* = 0.008), truncal/lower extremity injuries (HEM 60% vs. LEM 6%, *P* <0.001), neurosurgical procedures for the management of ICH (HEM 54% vs. LEM 36%, *P* = 0.003), and decreased Glasgow Coma Score on arrival (HEM 11.7 vs. LEM 14.2, *P* <0.001).

**Conclusion:**

FFs after HEM events were associated with severe and multifocal injuries. FFs after LEM events were associated with ICH, concussions, and cervical spine fractures. Mechanism-based screening strategies will allow for the appropriate detection and management of injuries that occur concomitant to FFs.

**Type of study:**

Retrospective cohort study.

**Level of evidence:**

Level III.

## Introduction

Dating back to the foundational work of Rene Le Fort, facial fractures (FFs) are recognized as an important source of morbidity after injury and are often associated with significant concomitant trauma [1]. Seemingly low-energy mechanisms (LEMs) such as assaults or ground-level falls can often lead to considerable craniofacial injuries, and high-energy mechanisms (HEMs) such as motor vehicle or bicycle crashes can leave clinicians managing major multisystem trauma alongside the FFs. Due to the innate biomechanics of the craniofacial bones, similar FF patterns can occur after a wide spectrum of traumatic forces, and so it is often times unclear what diagnostic tests are warranted in order to detect concomitant injuries and guide management decisions [2]

There have been a limited number of studies comparing the differences in fracture patterns, concomitant injuries, and management of patients with FFs following an LEM or HEM event. Prior studies describing patient outcomes after a FF often grouped heterogeneous mechanisms of injury (MOIs) together when evaluating mortality rates, airway management needs, or rates of intracranial hemorrhage (ICH) [3–7]. Grouping together both high-energy and low-energy MOIs, however, risks missing potential differences in injuries and outcomes between the two groups. A more granular understanding of injury patterns and outcomes can be obtained with mechanism-specific comparative analyses and, in an era when utilizing evidence-based screening protocols are of paramount importance, can better guide imaging and management decisions [8, 9]. In order to better differentiate the injuries and outcomes in patients with FFs after various MOIs, we evaluated fracture patterns, concomitant injuries, procedures required, timing of procedures, and outcomes in patients presenting with FFs after LEMs and HEMs.

## Methods

After Institutional Review Board approval, we conducted a retrospective review of patient records maintained in our trauma registry at the Santa Barbara Cottage Hospital (SBCH). SBCH is an academic teaching hospital, regional referral center, and an American College of Surgeons verified Level-1 Trauma Center in Santa Barbara, CA. We identified all adult patients aged 18–55 years old evaluated at SBCH for facial fractures (ICD-9 Codes 802.0-802.9 and 804.0-804.9, ICD-10 Codes S02.2-S02.9) from January 2013 to April 2019. Patients were then classified into LEM or HEM, defined by MOI (Table 1). Statistical analysis was performed using R Version 3.5.1 [10]. Patient demographics, comorbidities, FF region, concomitant injuries, procedures performed, and mortality rates were compared using Fisher’s exact test of independence. FFs were divided into upper face (supraorbital rim, orbital roof, frontal bone, frontal sinus), midface (nasal, maxillary, zygomatic, orbital wall, ethmoid, dentoalveolar bones), and those involving the mandible. Injury severity score (ISS), hospital length of stay (LOS), intensive care unit (ICU) LOS, and number of days from admission until operation for patients that underwent surgery were compared using an independent two-sample Mann-Whitney *U* test.

**Table 1:** Mechanisms of injury

**Low energy (*n* = 90)**	
Assault (%)	64 (65.3)
Ground-level fall (%)	14 (14.3)
Bicycle/skateboard crash (%)	12 (12.2)
Sports-related (%)	5 (5.1)
Work-related (%)	2 (2.0)
Self-inflicted (blunt head injury) (%)	1 (1.0)
**High energy (*n* = 25)**	
Fall from height >20 feet (%)	13 (52.0)
Motor vehicle crash (%)	5 (20.0)
Auto vs. pedestrian (%)	4 (16.0)
Motorcycle crash (%)	2 (8.0)
Gunshot to face (%)	1 (4.0)

## Results

We identified 123 patients with FFs, 98 (80%) were due to LEMs, and 25 (20%) were from HEMs. There were no significant differences in sex, age, history of osteoporosis, pre-injury steroid or tobacco use, mean calcium or albumin levels, or anticoagulant use. The most common LEMs were assault (65%), ground-level falls (14%), and bicycle/skateboard crashes (12%). The most common HEMs were falls from height > 20 feet (6 m) (52%), motor vehicle crashes (20%), and auto-versus pedestrian crashes (16%). Over half (54%) of LEM patients tested positive for ethanol in contrast to only 22% of HEM patients (*P* = 0.024). HEM patients had a longer overall LOS (HEM 14 days vs. LEM 4 days, *P* <0.001), longer ICU LOS (HEM 12 days vs. LEM 4 days, *P* = 0.011), greater burden of injury based on ISS (HEM 16.8 vs. LEM 7.5, *P* <0.001), and subsequently higher ICU admission rates (HEM 60% vs. LEM 13%, *P* <0.001). Table 2 summarizes patient demographics, comorbidities, and outcomes.

**Table 2:** Patient demographics, comorbidities, and outcomes

	**Energy mechanism**		***P*** value
	**Low (*n* = 98)**	**High (*n* = 25)**	
Age (SD)^1^	32.4 (12.6)	4.9 (13.1)	0.489
Number of males (%)^2^	87 (88.8)	19 (76.1)	0.18
History of osteoporosis (%)^2^	0/97 (0)	0 (0)	NA
Pre-injury steroid use (%)^2^	1/97 (1)	0 (0)	1
Pre-injury tobacco use (%)^2^	26/97 (27)	5 (20)	0.661
Pre-injury anticoagulant use (%)^2^	0 (0)	2 (8)	0.053
Calcium levels (SD)^1^	9.0 (0.7), *n* = 95	9.0 (0.6)	0.819
Albumin levels (SD)^1^	4.3 (0.4), *n* = 95	4.3 (0.7)	0.211
Positive ethanol (%)^2^	44/82 (54)	5/24 (22)	0.012*
Hospital LOS—days (SD)^1^	4 (4), *n* = 67	14 (12), *n* = 23	< 0.001*
ICU LOS—days (SD)^1^	4 (3), *n* = 13	12 (10), *n* = 15	0.011*
ICU admittance (%)^2^	13 (13)	15 (60)	< 0.001*
Needing ventilator (%)^2^	8 (8)	12 (48)	< 0.001*
Days on ventilator (SD)^1^	4.7 (6.5)	10.7 (7.7)	0.081
ISS (SD)^1^	7.5 (6.3)	16.8 (9.8)	< 0.001*
GCS (SD)^1^	14.2 (2.1)	11.7 (4.2)	0.002*
Mortality (%)^2^	1 (1)	1 (4)	0.367

^1^Two-sample Mann-Whitney *U* test

^2^Fisher’s exact *T* test

FF patterns were compared, and we found no differences in rates of upper face (HEM 24% vs. LEM 13%, *P* = 0.217), midface (HEM 84% vs. LEM 63%, *P* = 0.057), mandible (HEM 20% vs. LEM 38%, *P* = 0.105), Le Fort (HEM 12% vs. LEM 3%, *P* = 0.098), or bilateral fractures (HEM 32% vs. LEM 17%, *P* = 0.161). Facial surgeries were required in 40% of LEM patients and in 28% of HEM patients (*P* = 0.357). The most common procedures in each group were to repair mandible fractures (HEM 43%, LEM 85%). Nearly one quarter of mandible fractures in the LEM group were managed solely with intermaxillary fixation (IMF); in contrast, none of the FFs in the HEM group were managed using IMF. The median hospital day when facial procedures were performed was similar between the two groups (HEM day 2 vs. LEM day 1, *P* = 0.086).

Computed tomographic angiography (CTA) rates were lower in the LEM group (HEM 36% vs. LEM 5%, *p*<0.001), but no cerebrovascular injuries were found in either group. Patients with HEM injuries presented with lower mean Glasgow Coma Scores (GCS) (HEM 11.7 vs. LEM 14.2, *P* = 0.002), higher rates of ICH (HEM 52% vs. LEM 15%, *p*<0.001), and more often required neurosurgical procedures (HEM 54% vs. LEM 36%, *P* = 0.003). HEM patients had higher rates of torso/extremity injuries (HEM 64% vs. LEM 7%, *P* = 0.045), and specifically, higher rates of cervical spine fractures (HEM 12% vs. LEM 0%, *P* = 0.008), thoracic injuries (HEM 56% vs. LEM 5%, *p*<0.001), abdominal injuries (HEM 8% vs. LEM 0%, *P* = 0.04), and pelvic lower extremity injuries (HEM 24% vs. LEM 1%, *P* = 0.034). The HEM group had higher rates of surgical procedures of any kind (HEM 72% vs. LEM 49 %, *P* = 0.045). There was no difference in mortality between the groups (HEM 4% vs. LEM 1%, *P* = 0.367). Table 3 summarizes our injury, procedure, and outcome data. Table 4 summarizes the FF procedures performed.

**Table 3:** Facial fracture location, associated injuries, and procedures

	**Energy mechanism**		***P*** value
	**Low (*n* = 98)**	**High (*n* = 25)**	
**Fracture location**			
Upper third (%)^1^	13 (13)	6 (24)	0.217
Midface (%)^1^	62 (63)	21 (84)	0.057
Mandible (%)^1^	37 (38)	5 (20)	0.105
Bilateral (%)^1^	17 (17)	8 (32)	0.161
Le Fort (%)^1^	3 (3)	3 (12)	0.098
Multiple facial fractures^1^	45 (46)	15 (60)	0.264
**Associated injuries**			
Any head or neck injury (%)^1^	35 (36)	17 (68)	0.265
Intracranial hemorrhage (%)^1^	15 (15)	13 (52)	< 0.001*
Cerebrovascular (%)^1^	0 (0)	0 (0)	NA
Concussion (%)^1^	33 (34)	14 (56)	0.064
Cervical spine fracture (%)^1^	0 (0)	3 (12)	< 0.001*
Any torso or extremity injury (%)^1^	7 (7)	16 (64)	0.0453*
Thoracic (%)^1^	5 (5)	14 (56)	< 0.001*
Abdominal (%)^1^	0 (0)	2 (8)	0.040*
Pelvic/lower extremity (%)^1^	1 (1)	6 (24)	< 0.001*
**Procedures**			
CTA^2^ performed (%)^1^	5 (5)	9 (36)	< 0.001*
Any procedure (%)^1^	48 (49)	18 (72)	0.045*
Neurosurgical procedure (%)^1^	5 (5)	7 (28)	0.002*
Facial surgery required (%)^1^	39 (40)	7 (28)	0.357

^1^Fisher’s exact *T* test

^2^Computed tomography angiography

**Table 4:** Procedure summary

	**Low energy (*n* = 39)**	**High energy (*n* = 7)**
IMF/closed only (%)	8 (21)	0 (0)
IMF mandible ORIF unilateral (%)	11 (28)	1 (14)
IMF mandible ORIF bilateral (%)	7 (18)	2 (29)
Mandible ORIF unilateral (%)	3 (8)	0 (0)
Mandible ORIF bilateral (%)	4 (10)	0 (0)
Zygoma ORIF (%)	2 (5)	1 (14)
Orbital floor ORIF (%)	2 (5)	0 (0)
Zygoma and orbital floor (%)	1 (3)	0 (0)
Le Fort ORIF (multiple) (%)	0 (0)	0 (0)
Le Fort ORIF (multiple) and IMF (%)	1 (3)	2 (29)
Frontal sinus ORIF (%)	0 (0)	1 (14)

*IMF* intermaxillary fixation, *ORIF* open reduction internal fixation

## Discussion

FFs can occur after a wide variety of mechanisms. Injuries concomitant to FFs can often be significant, and complicate clinical management and patient recovery after trauma. Our analysis of 123 patients with FFs after both high and low MOIs showed similar fracture pattern rates and need for facial operations, but significant differences in associated injuries and the complexity of care required to manage them.

When comparing the types of fracture patterns that occurred between the HEM and LEM groups, we found no significant differences in fracture rates based on facial region, bilaterality, or those with Le Fort architecture. Additionally, we found no difference in facial surgery rates or time to facial surgery. Prior to this analysis, we would have predicted that HEM patients would have more severe facial injuries, and subsequently would require operative management more often. Furthermore, we suspected that definitive facial repair would be delayed until later in the hospital course as higher priority injuries were managed first. Analysis of our data, however, suggests that none of these conjectures are valid. Though the HEM group had a higher burden of injury and an overall higher need for procedures, these same findings are not reflected in the types of injuries, timing, or procedures needed to manage facial injuries. The underlying biomechanics of the facial bones lend themselves to predictable fracture patterns, but whether highly variable MOIs as seen in our analysis generate traumatic forces similar enough to fracture these bones similarly is unclear; our data suggests this may be the case, but clearly a dedicated study with a larger sample size is warranted to further explore these findings [2].

Over half of our LEM patients tested positive for ethanol in contrast to only 22% of our HEM patients. Prior studies have recognized ethanol use as a common feature of patients at the time of facial trauma, but the higher prevalence in patients with FFs after LEMs compared to HEMs has not been reported [11, 12]. Given the two most common MOIs for the LEM group were assaults and ground-level falls, our findings further implicate ethanol use as an important factor in these MOIs, and may offer an opportunity for targeted injury-prevention strategies. Our LEM patients had very low rates of injuries “below the clavicles” (only 7% of patients), but 15% had ICHs, and 34% had concussion symptoms. Over 13% of LEM patients required ICU admission with a mean ICU LOS of 4 days. These findings speak to the possibilities of serious neurological injuries associated with FFs after seemingly minor MOIs. This is particularly true in the setting of concomitant ethanol use at the time of injury which can potentially cloud patient evaluation and should serve to heighten the need to aggressively screen these patients for intracranial, craniofacial, cervical, and cerebrovascular injuries. Given the low prevalence of torso/extremity injuries, routine radiological imaging may not be necessary in patients with FFs after LEM unless a clear indication exists; validation of our findings with a multicenter analysis is warranted.

Patients suffering from FFs after HEMs often had severe concomitant injuries complicating their care. Fifty-two percent had ICHs, nearly half of whom required neurosurgical procedures to manage these injuries. In addition to these neurological injuries, HEM patients had nine times the rates of torso and extremity injuries compared to LEM patients (HEM 64% vs. LEM 7%, *P* = 0.045). Correspondingly, HEM patients had nearly double the mean ISS, were admitted to the ICU five times as often, and had an average ICU LOS 8 days longer than their LEM counterparts. Patients with FFs from HEMs were considerably more injured and required longer and more complex medical care. Given these findings, aggressive detection of the head, neck, torso, and extremity injuries and subsequent early transfer to an advanced trauma center for management are warranted.

While there was no significant difference in fracture patterns or FF procedure rates between the two groups, the profile of FF procedures differed. Procedures performed in the LEM population were almost exclusively for the treatment of mandibular fracture (85%) compared to only 45% of facial procedures performed in the HEM patients. Unfortunately, given the small number of procedures performed, deeper analysis of differences in FF procedures performed is difficult in our data. Corresponding to the higher rates of non-craniofacial injuries, the HEM group required higher rates of procedures to manage these injuries. Interestingly, despite the greater injury burden, procedures required, and longer hospital course, we found no differences in mortality between the two groups. This finding suggests that, despite the higher burdens of injury and more complex hospital courses in these patients, their life-threatening injuries were appropriately detected and managed. These findings support the need for proactive detection of concomitant injuries and the subsequent multidisciplinary management of these patients at high-quality trauma systems.

Our study has several important limitations. This was a retrospective review of a prospectively maintained trauma database and so is vulnerable to errors in data coding and retrieval. SBCH is the only ACS-verified Level-I Trauma Center for the Santa Barbara County region, and though the majority of FFs in our area are referred to us, we were not able to capture those patients to whom referral was not offered, or those refusing transfer from remote hospitals; the impact these patients have on our understanding of FFs in our region is not known. We were not able to evaluate other intoxicants beyond ethanol in our review; clearly, this information would deepen our understanding of the role that intoxication may play in these injuries and may serve as a topic for dedicated study. Only seven HEM patients required operative fixation of their FFs in contrast to 39 patients in the LEM. These small numbers severely hampered our ability to conduct deeper analysis into differences in the types of procedures required, and we are unable to draw conclusions to this question. We did not independently audit the treatment decisions made (operative vs. nonoperative, nor the type of operation performed), and as such, we are unable to comment on the overall absolute rates of operative intervention. The majority of fractures in both groups were managed nonoperatively, and no difference in the relative rates of operative intervention was found; we are unable to draw any further conclusions in regards to the rates of operations in our study population. Lastly, this was a single-institution study of FFs occurring in the Santa Barbara County region; our data analysis is limited by the small patient population, and so leaves us vulnerable to type II statistical error. The body of literature regarding FFs would benefit from a dedicated multicenter analysis.

## Conclusion

FFs after HEM events were associated with severe and multifocal injuries. FFs after LEM events were associated with ICH, concussions, and cervical spine fractures. Mechanism-based screening strategies will allow for the appropriate detection and management of injuries that occur concomitant to FFs.

## Data Availability

The datasets generated for the current study are not publicly available as they contain health information protected under the U.S. Health Insurance Portability and Accountability Act of 1996 but are available upon reasonable request.

## Abbreviations

FF: Facial fracture
HEM: High-energy mechanism
LEM: Low-energy mechanism
MOIs: Mechanisms of injury
ICH: Intracranial hemorrhage
SBCH: Santa Barbara Cottage Hospital
ISS: Injury Severity Score
LOS: Length of stay
ICU: Intensive care unit
HD: Hospital day
GCS: Glasgow Coma Scores
